# A population survey on beliefs around cervical cancer screening: determining the barriers and facilitators associated with attendance

**DOI:** 10.1186/s12885-022-09529-w

**Published:** 2022-05-09

**Authors:** Gaby Judah, Faisal Dilib, Ara Darzi, Sarah Huf

**Affiliations:** Department of Surgery and Cancer, Imperial College London, St Mary’s Campus, Praed Street, London, W2 1NY UK

**Keywords:** Cervical cancer screening, Theoretical Domains Framework, Barriers, Facilitators, Behaviour change, Survey, Cancer screening behaviour, Screening attendance

## Abstract

**Background:**

Cervical screening saves approximately 5000 lives annually in England. However, screening rates have been falling continuously, and coverage in London is particularly low (64.7%). While demographic predictors of uptake have been well researched, there has been less thorough investigation of the individual barriers and facilitators which predict cervical screening attendance. Understanding modifiable factors influencing attendance can guide the design of effective interventions to increase cervical screening uptake. The aim of this study was to understand the demographic, and individual factors associated with self-reported attendance at cervical screening in London.

**Methods:**

The study used an online survey of 500 women in London (June-July 2017). The survey included self-reported measures of past attendance, demographic variables (including age, household income, ethnicity), past experience variables, and individual variables (list of potential barriers and facilitators developed based on the Theoretical Domains Framework and existing literature, which included: environmental context and resources, perceived risk, anticipated pain/embarrassment). Participants were categorised into regular attenders and non-regular attenders. Backwards stepwise logistic regression investigated the barriers and facilitators predicting past attendance. Demographic variables with significant differences between regular and non-regular attenders were added to the final regression model.

**Results:**

Of women who had previously been invited (*n* = 461, age range: 25–65), 34.5% (*n* = 159) were classified as non-regular attenders, and 65.5% (*n* = 302) as regular attenders. The individual barriers and facilitators predicting attendance were: cervical screening priority, memory, environmental context and resources, and intention. The only demographic variables related to regular attendance were relationship status (married/civil partnership having higher rates than single) and higher household income. Relationship status was not significant when adjusting for barriers and facilitators. Those who have ever been sexually active or who have had an STI in the past were significantly more likely to be regular attenders.

**Conclusions:**

The study shows the importance of individual barriers and facilitators in predicting self-reported cervical screening attendance. Household income was the only significant demographic variable when combined with the individual variables. Interventions targeting priority, memory, and practical barriers affecting environmental context may be expected to be effective an increasing attendance.

**Supplementary Information:**

The online version contains supplementary material available at 10.1186/s12885-022-09529-w.

## Background

Population level cervical screening (CS) can significantly reduce cervical cancer morbidity and mortality [1] through early detection and treatment. However, regular participation in CS is key as it is estimated that 83% of cancer deaths could be avoided if all women eligible for CS were screened regularly (whenever invited) [2]. A recent review of adult screening programmes in England highlighted that cervical screening rates in the UK have been falling in the last decade [3]. Age-appropriate coverage in the UK fell from 75.7% in 2010/11 to 71.9% in 2018/19 [4]. (Coverage is defined as the percentage of eligible women screened within a specified timeframe, which in the UK is 3.5 years for women aged 25 to 49, and 5.5 years for women aged 50–64). Coverage in 2018 was lower in younger age-groups (25–49 years, 69.1%) than older age-groups (50–64 years, 76.2%) [4]. Despite regional differences, no area reached the national target of 80% coverage, and London had one of the lowest coverage rates (64.7%). These low rates are seen despite evidence that 90% of people in Britain [5] have very positive attitudes towards participation in cancer screening. Furthermore, 17% of samples tested in 2017/2018 were ‘opportunistic’ samples, meaning that they were taken when women presented to primary care for another reason, suggesting that many women are still not making appointments when invited for cervical screening [3].

Several papers have reported relationships between demographic factors and cervical screening attendance. There is evidence that in the UK, CS rates are low in more deprived areas, ethnic minority groups, and the youngest age group (25–29 years) [3, 6]. Survey data from Great Britain indicates that screening uptake is higher in more educated women [6]. From a large survey study in Denmark, non-participation in screening (of passive non-attenders, rather than those who actively unsubscribed) was more common in women who had basic education, low income, were unmarried, from less developed countries, had 4 or more children, or history of obesity [7]. In Norway, attendance was positively associated with being married or cohabiting, being a non-smoker, and having given birth [8]. However, demographic predictors do not lend themselves to behaviour change interventions directed at improving cancer screening.

Recently published work [9] showed that text message reminders can significantly increase CS uptake, and that messages based on different behaviour change techniques varied in their ability to improve CS participation. Of the messages tested, a neutral reminder and a GP endorsed message were found to be the most effective. However, basing interventions on observed barriers to CS may lead to interventions with greater impact on behaviour. Awareness of individual barriers and facilitators associated with cervical screening attendance will help guide the design of more effective interventions to increase screening uptake.

A number of studies have investigated barriers and facilitators for CS attendance, however often these have not linked their findings to actual or reported attendance. For example by only recruiting women who were attending or who had recently attended cervical screening [10, 11], or by only measuring intention to attend cervical screening rather than self-reported or objectively measured attendance [12]. This is important, as not all factors reported as strong barriers to CS are actually associated with screening attendance. In a face-to-face survey study in England of 580 randomly selected women aged 26–64, embarrassment was the most frequently reported barrier (29%) [13], however was not associated with being overdue for screening. Similarly, no association was found between being overdue for screening and the commonly reported barriers of: worrying about the test being painful; previous bad experience; being scared of what the test might find; and not feeling at risk of cervical cancer. However, this study did find a relationship between being overdue for screening and more practical barriers, such as: not getting round to going for screening straight away; or difficulties with getting a convenient appointment time. A systematic review of qualitative studies of experiences of CS in countries with a national screening programme found that practical barriers affected whether intentions to be screened were translated into actual screening attendance [14], however, women who had never attended screening were poorly represented in the samples.

A Dutch study of 200 women, based on the Integrated Model for Behaviour Change, found that, compared to attenders, non-attenders estimated the risk of getting cervical cancer as lower, reported fewer positive role models and social support, and thought it would be harder to overcome barriers including making an appointment, and finding time to attend [15]. Non-attenders reported more cognitive barriers (e.g. seeing participation as more time-consuming and worrying about the result) and affective barriers (e.g. greater insecurity, anxiety and shame from the test itself). Factors independently associated with attendance were subjective norm, barrier self-efficacy, and (with a small effect size) ambivalence towards screening. However, it is not clear whether the findings from this small study generalise to the UK context.

Except the small study by Knopps-Dullen and colleagues [15], the majority of existing work on barriers to cervical attendance has not been based on a theory or model of behaviour, and so may not comprehensively assess barriers to attendance. The present study measured predictors of CS attendance in women in London, using a survey based on the Theoretical Domains Framework (TDF) [16]. The TDF was developed to support the design of interventions to change behaviour through identification of barriers and facilitators that need to be addressed. The TDF is a comprehensive framework combining domains from 33 different theories of behaviour and 128 theoretical constructs, thus increasing the likelihood that all potential behavioural predictors will be assessed. In addition, the TDF can be cross referenced to the Behaviour Change Wheel [17], or the Theory and Techniques Tool [18], and subsequently the Behaviour Change Techniques Taxonomy [19], which provide a systematic way to map TDF barriers or facilitators to techniques to change behaviour which are supported by theory and evidence. Using the TDF to comprehensively identify predictors of CS is a novel and valuable approach, which can support the design of a behavioural intervention which effectively addresses the observed behavioural barriers and facilitators to cervical screening uptake, in a systematic and theory driven way.

The aim of this study was to understand the demographic factors and individual barriers and facilitators associated with self-reported attendance at cervical screening in London. Using a survey we aim to establish the strongest barriers and facilitators predicting past CS attendance.

## Methods

### Participants

Women aged 24–65 in London were invited to participate in the online survey through a third party market research company (Bilendi, www.bilendi.co.uk). Women younger than 24 or older than 65, or those who had undergone a hysterectomy were excluded. Data was discarded from those who did not complete the whole survey. Fifty women completed the pilot survey. Following item removal (see “Barriers and facilitators” section”), a further 450 women completed the survey, leading to a total sample of 500. The data was collected from June-July 2017, using online and offline recruitment including advertising in public places and public domains online. Participants were renumerated with reward points by Bilendi (150 points) which can be collected then exchanged for items and services.

### Measures

The survey was developed based on a review of commonly reported predictors of screening in the literature. The survey comprised outcome variables (past attendance), and demographic, past-experience and individual barriers and facilitators.

Past attendance was measured using the mean of two items (“In the past, when invited, I have attended a smear test” and “In the past, when invited, I have always made an appointment for my smear test”) with responses on a 5-point scale (always, often, sometimes, rarely, never). As past attendance scores were positively skewed, this mean score was split into a binary variable with those who scored 4.5–5 classed as regular attenders, and those who scored 4 and below classed as non-regular attenders. Those who responded “often” to both of the past-attendance questions were therefore classed as non-regular attenders. This is because risk of mortality is greater in those who do not always attend screening [2], therefore understanding reasons for not always attending will inform the design of interventions to promote consistent attendance to screening, in order to reduce mortality.

The questionnaire included the following demographic variables: age, ethnicity, highest level of education, total household income, employment status, relationship status and whether English was their first language.

The questionnaire included items on aspects of past experience which may affect CS attendance. These were: having had a close relative/friend diagnosed with cervical cancer; ever having had another type of cancer; every having been sexually active; ever having had a sexually transmitted infection (STI), past physical, psychological or sexual abuse. At the request of the stakeholders within the screening service, civic engagement was also measured to see whether this is related to screening attendance, by asking whether they had voted in the last general election, or the Brexit referendum. Each was measured using a single item, with responses of Yes/No/I don’t know.

### Barriers and facilitators

The constructs measured were based on the Theoretical Domains Framework (TDF) [16], and informed by findings from the literature. The constructs are shown in Table 1, linked to the domains from the TDF. Construct contained between two and five items, with responses on a 7-point scale (1 = strongly disagree, 7 = strongly agree). Some items were reverse scored.

**Table 1:** Survey constructs, mapped onto the Theoretical Domains Framework

TDF Domain	Survey Constructs (number of items)
Knowledge	1. Knowledge of cervical cancer (2) 2. Knowledge of cervical cancer screening programme (3) 3. Knowledge of benefits of screening (5) 4. Knowledge of cervical cancer risk factors (3)
Skills	Not relevant
Social/professional role and identity	5. Perceived risk (2)
Beliefs about capabilities	6. Perceived behaviour control (2)
Optimism	7. Pessimism—emotional consequences of potential results (2)
Beliefs about Consequences	8. Value (3) 9. Belief about test effectiveness/specificity (2)
Reinforcement	10. Reassurance (2) 11. Previous negative experience (2)
Intentions	12. Intention (2)
Goals	13. Health priority (2) 14. Cervical screening priority (2)
Memory, attention and decision processes	15. Memory (2)
Environmental context and resources	16. Environmental context and resources (4)
Social influences	17. Social norms – descriptive (2) 18. Social norms – injunctive peers (2) 19. Social norms – healthcare professionals (2)
Emotion	20. Anticipated pain/embarrassment (3)
Behavioural regulation	21. Behaviour regulation and planning (2)

A pilot survey was used to reduce the number of items, using Cronbach’s alpha analysis to test internal consistency [20]. Nine items were removed following the pilot, leaving 51 items for barriers and facilitators. The full list of items, grouped by construct, and the Cronbach’s alpha scores from the full sample, are shown in Additional file 1.

### Ethics

After reading the information about the study, participants indicated their informed consent to take part by ticking a box on the information page at the beginning of the survey. Ethical approval was received from Imperial College Research Ethics Committee (ICREC reference: 17IC3937). All study methods were performed in accordance with relevant guidelines and regulations.

### Analysis

The association between regular versus non-regular attendance, and the demographic and past experience variables, were tested using Chi squared tests.

Univariate group comparisons between regular and non-regular attenders on the barriers and facilitators were made using t-tests, adjusted so as not to assume equal variances when necessary (when Levene’s test for equality of variance was significant). An exploratory logistic regression (backwards stepwise) was conducted to see which constructs independently predicted past attendance, in order to assess all potential barriers or facilitators to the behaviour. Where a TDF domain was subdivided into multiple constructs, each construct was treated individually as an independent variable in the analysis. Demographic and past experience variables significantly associated with attendance from the Chi Squared tests were added to the final model predicting attendance from the stepwise regression. For all analyses, the alpha level was set as 0.05. The analysis was conducted using IBM SPSS version 25.

## Results

### Sample

The sample of 500 women had a mean age of 42.5 years (SD = 10.7 years). The sample was predominantly white (65.5%). The sample demographics can be found in Table 2.

**Table 2:** Sample demographics, and significance testing of sample of women in London (June-July 2017)

	***N***	**Number regular attenders**	**% regular attenders**	**Pearson Chi-Square**	***p* value**
**Age (*N* = 461)**				1.586	.453
24–34	118	72	61.0%		
35–49	201	133	66.2%		
50–64	142	97	68.3%		
**Ethnicity (*N* = 414) **^**a**^				3.738	.443
Did not disclose	20	10	50%		
White	290	190	65.5%		
Biracial	14	10	71.4%		
Asian	36	27	75.0%		
Black	50	32	64.0%		
Any other ethnic group	4	1	25.0%		
**Employment status (*N* = 461)**				7.343	.196
Employed, working full-time	227	149	65.6%		
Employed, working part-time	95	67	70.5%		
Not employed, looking for work	35	22	62.9%		
Not employed, NOT looking for work	51	27	52.9%		
Retired	25	20	80%		
Disabled, not able to work	28	17	60.7%		
**Household Income (*N* = 461)**				11.823	.066
Less than £24,999	128	79	61.7%		
£25,000—£34,999	78	53	67.9%		
£35,000—£49,999	69	36	52.2%		
£50,000—£74,999	65	49	75.4%		
£75,000—£99,999	32	23	71.9%		
£100,000 or more	24	19	79.2%		
Do not wish to disclose	65	43	66.2%		
**Relationship status (*N* = 461)**				19.304	.002
Single	160	86	53.8%		
Single, cohabiting with a significant other/domestic partnership	77	49	63.6%		
Married/In a civil union	173	132	76.3%		
Separated	17	11	64.7%		
Divorced	25	18	72.0%		
Widowed	9	6	66.7%		
**Education (*N* = 461)**^**b**^				0.685	.953
0-level/GCSE	71	46	64.8%		
A-level/Secondary school graduate	63	40	63.5%		
Trade/Technical/Vocational qualification	73	48	65.8%		
Bachelor’s degree	157	106	67.5%		
Masters/other postgraduate degree	86	54	63.0%		
Other, please specify	11	8	72.7%		
**English first language? (*N* = 461)**				1.140	.286
Yes	361	232	64.3%		
No	100	70	70%		

^a^ A single respondent of ‘Arab’ in the ethnicity categories was classified as ‘Asian’, and the categories of Other and Biracial were combined to allow the Chi Squared test to be conducted (with no expected values < 5)

^b^ The ‘other’ category was removed for performing the chi squared test

Of the 461 participants who reported having been invited to cervical screening in the past, mean attendance scores were 4.26 (SD = 1.17) (maximum score = 5), with 159 participants (34.5%) classified as non-regular attenders and 302 (65.5%) as regular attenders.

### Survey validity

Survey domains were tested for internal consistency using Cronbach’s alpha. Alpha for past attendance was 0.96. All barriers and facilitators had alpha scores above the recommended threshold of 0.7, except “Beliefs about test effectiveness/specificity which had a score of 0.574 (see Additional file 1). As it was not possible to remove items to improve this score, the domain was still included in the analysis.

### Association between attendance and demographic factors

Demographic differences between regular and non-regular attenders are shown in Table 2. Only relationship status showed significant differences. Women in the ‘Single’ category had lowest levels of regular attendance (53.8%) and women in the ‘Married/civil partnership’ group had the highest levels (76.3%). The test for household income was significant at the *p* < 0.1 level (${\chi }^{2}$ = 11.823, *p* = 0.066). When annual household income was split into a binary category for under £50,000 and £50,000 and over, there were significant differences (${\chi }^{2}$ = 7.399, *p* = 0.007): 61.1% of the low income group were regular attenders, versus 75.2% of the high income group.

## Impact of past experience on attendance

As shown in Table 3, those who have ever been sexually active are significantly more likely to attend screening (${\chi }^{2}$ = 15.307, *p* < 0.001). Those who have had an STI in the past are also more likely to attend screening (${\chi }^{2}$ = 4.153, *p* = 0.042).

**Table 3:** Relationship between past sexual history and cervical screening attendance

	***N***	**Number regular attenders**	**% regular attenders**	**Pearson Chi-Square**	***p* value**
**Past sexual activity**				15.307	< .001
Yes	399	276	69.2%		
No	58	25	43.1%		
**Past STI (Sexually transmitted infection)**				4.153	.042
Yes	71	54	76.1%		
No	384	244	63.5%		

There was no difference found in regular attendance between those who had had a close family member or friend diagnosed with cervical cancer, respectively (${\chi }^{2}$ = 3.390, *p* = 0.184) and (${\chi }^{2}$ = 1.693 *p* = 0.445). There was also no difference in levels of regular attendance in levels of regular attendance in women who have ever experienced physical, psychological or sexual abuse, respectively (${\chi }^{2}$ = 0.303, *p* = 0.582), (${\chi }^{2}$ = 1.189, *p* = 0.276) and (${\chi }^{2}$= 0.228, *p* = 0.672).

Considering the variables measuring civic engagement, there was no difference in levels of regular attendance based on whether or not women voted in the last general election (${\chi }^{2}$ = 0.393, *p* = 0.531), or whether they voted in the Brexit referendum (${\chi }^{2}$ = 0.000, *p* = 0.999).

### Summary of barriers and facilitators

The means of the barriers and facilitators for participants classed as regular and non-regular attenders, and group comparisons, are shown in Additional file 2. Most domains showed highly significant group differences. The only domains that did not were knowledge of cervical cancer risk factors, belief about test effectiveness/specificity, perceived behavioural control, knowledge of cervical cancer, and emotional consequences of potential results (the latter two constructs had *p* < 0.1). The constructs with the largest differences (all with lower scores for non-regular attenders) were cervical screening priority (t(220) = 18.78, *p* < 0.001), intention (t(194) = 14.73. *p* < 0.001), planning (t(272) = 12.75, *p* < 0.001) and memory (t(262) = 16.25, *p* < 0.001).

### Predicting past attendance

The regression included only those participants who reported having been invited for a smear test in the past (*N* = 461). Relationship status, income above or below £50,000, past sexual activity and past STI were added to the final model of the barriers and facilitators. Relationship status was not significant (*p* = 0.555) so was removed from the model, as were past sexual activity (*p* = 0.347) and past STI (*p* = 0.227). The final model is shown in Table 4. Cervical screening priority was the strongest predictor of past attendance (B = 1.089, *p* < 0.001), followed by memory (B = 0.941, *p* < 0.001). Other predictors were environmental context and resources (B = 0.501, *p* < 0.001), intention (B = 0.480, *p* = 0.005) and income (B = 0.946, *P* = 0.018). Social norms – descriptive, and social norms – healthcare professionals were negatively related to being a regular attender, but did not reach significance at the *p* < 0.05 level (*p* = 0.059 and *p* = 0.060 respectively).

**Table 4:** Backwards stepwise logistic regression predicting cervical screening attendance

	**Unstandardized Coefficients**		**Odds Ratio**	**Sig**	**95.0% Confidence Interval for EXP(B)**	
	B	Std. Error			Lower Bound	Upper Bound
**(Constant)**	-11.809	1.521	0.000	< .001		
**Cervical Screening Priority**	1.089	.195	2.972	< .001	2.028	4.355
**Social Norms – descriptive**	-0.308	.163	0.734	.059	.534	1.012
**Social norms – healthcare professionals**	-.379	.201	0.685	.060	.461	.1.016
**Memory**	.941	.185	2.564	< .001	1.783	3.687
**Environmental context and resources**	.501	.126	1.651	< .001	1.289	2.113
**Intention**	.480	.169	1.616	.005	1.160	2.251
**Income**	.946	.402	2.575	.018	1.172	5.656

Nagelkerke R Square = .733, classification 88.1% correct

## Discussion

This survey study found that 34.5% of women who had previous been invited to screening reported being non-regular attenders. The strongest predictors of regular attendance were prioritising cervical screening, and memory. Other predictors were environmental context and resources, intention, and having a household income of £50,000 or over. No other demographic variables were found to significantly predict regular attendance, except that women who were married or in a civil partnership were more likely to be regular attenders than single women, but this was only found in the univariate comparison. Having ever been sexually active, and having had a STI in the past were related to higher rates of reported regular attendance in univariate comparisons, but likewise, these were not significant when included with individual barriers and facilitators.

The finding that prioritising cervical screening was the strongest predictor of reported past attendance is consistent with previous research. A UK survey found that an independent predictor of being overdue for screening was not getting round to it right away [13]. The importance of priority has also been highlighted in qualitative work [21], and another theory-based survey, which found that the extent to which women felt incapable of getting screened due to competing commitments (analagous to ‘cervical screening priority’ in this study) was associated with lower attendance [15]. Similarly, we found that memory was a significant predictor of regular attendance. This is consistent with other research, where forgetting to make an appointment was a main reason for non-attendance in a survey study of Dutch women [22], and not getting round to making an appointment was a commonly reported barrier in a study of Black women in London [23].

The importance of environmental context and resources has been commonly reported in other studies, with factors such as a lack of time or childcare being reported as a barrier to attendance [11, 24, 25], and being associated with lower attendance levels [13, 15]. Struggling to arrange a convenient appointment time was found to be an independent predictor of being overdue for screening [13].

Interestingly, emotional factors were not significant predictors of reported regular attendance. This is consistent with the findings by Waller et al., whereby embarrassment was cited as a barrier, but was not predictive of attendance [13]. However, in contrast, a survey study in Australia found that embarrassment and anxiety were related to past screening [26].

Social norms variables were not independent predictors of reported regular attendance. While univariate comparisons found higher scores for social norms (descriptive, injunctive peers, and from healthcare professionals) in regular attenders, in the regression analysis the descriptive norms variable, and the social norms or expectations from healthcare professionals tended towards being predictors of non-regular attendance (with *p* < 0.1). Social norms do not appear to be a commonly reported predictor of attendance in the literature. However, Knops-Dullens et al., found attenders had more positive role models, and social support to attend [15]. Yet this study only reported univariate comparisons between attenders and non-attenders, so the findings are comparable to the positive univariate relationship observed in the present study between social norm variables and being a regular attender. It could be that smear tests are something that are not commonly discussed, and therefore the impact of social norms on behaviour is inconsistent, or not as expected.

Household income level was the only demographic factor which was a significant predictor within the regression model with barriers and facilitators. This is consistent with a recent systematic review which found that household income is associated with CS uptake [27]. However, that review also identified that all included studies except one found a positive relationship between education and screening uptake, yet this was not observed in the present study. The finding from univariate comparisons that women who were married/in a civil partnership were more likely to report being regular attenders than single women is consistent with several other studies [7, 8, 13]. These demographic findings can be used to support the targeting of an intervention to those who are least likely to attend (e.g. lower household income, and potentially single women or those who have not had an STI), and the inclusion of relevant subgroups when co-designing any intervention.

In the present study, while the younger age group did have lower absolute levels of regular attendance than older groups, this difference was not significant. Previously, younger age has commonly been associated with lower attendance [8, 24], however, other studies report that those in an older age category (55–64) are more likely to be overdue for screening than a younger category (35–44) [3, 6, 13]. Therefore, there are conflicting findings regarding the relationship between age and attendance. In contract to expectations, we did not observe higher rates of non-regular attendance in ethnic minority groups [13]. This may indicate that our sample, particularly those from ethnic minority backgrounds, may not be fully representative. It is interesting that we did not observe differences in regular attendance in women who had experienced physical, sexual or psychological abuse.

The study findings can help inform interventions which target individual barriers most closely associated with attendance. For example, reminder-based interventions, which target the priority for making an appointment are likely to be effective. In addition, addressing environmental barriers, such as availability of transport and childcare, or facilitating booking appointments through provision of online scheduling or out-of-hours appointments, is likely to support increased screening attendance. The finding in univariate comparisons that those who have ever been sexually active, and have had a STI in the past had higher rates of reported regular attendance may suggest an intervention which emphasises the importance of attendance even if one has not previously had an STI, or many sexual partners, or which is targeted to people who may be in that category. However, it is interesting that these variables were no longer significant when included in a model with individual barriers and motivators. Future research could consider the differences in barriers between higher and lower income groups, to ensure that interventions are likely to reduce screening inequalities.

### Study limitations and strengths

This was a large survey of a representative population in London. The income levels (45% of the sample with household income under £35,000) are representative of the London population [28], as are education levels (45% having a vocational degree or lower) [29]. The proportion classed as regular attenders (65.5%) is similar to London cervical screening coverage rates (64.7%) [3]. The survey was based on a comprehensive framework of behavioural predictors [16], and a review of barriers reported in the literature. The study quantitatively shows the predictors of attendance of a large representative sample, and the findings can be used to inform the design of interventions to improve CS uptake.

This study has some limitations. Attendance measurements were self-reported, rather than from objective attendance data. As the survey was conducted online and in English, it is only representative of the online, English-speaking population. This may explain why the percentage of participants of non-White ethnicity (26%) was lower than that found in London from the 2011 census, where 40.2% identified with being from an Asian, Black, Mixed, or Other ethnic group [30]. Therefore, certain segments of the population may not be well represented. In addition, there may be selection bias, in that women who have previously attended cervical screening may have been more likely to participate in a survey about cervical screening than non-attenders. However, the proportion classed as regular attenders (65.5%) is in line with London coverage rates for cervical screening (64.7%) [3]. Another key limitation is that the findings may have limited generalisability outside the London online population, or at least outside a UK urban setting. The use of backwards stepwise regression also has limitations, including a bias towards regression coefficients appearing larger, and p values appearing smaller. However, this approach was selected given that this was an exploratory analysis using a large yet comprehensive set of potential predictors of behaviour.

## Conclusions

Future work should establish whether the barriers and facilitators which predict self-reported attendance, can inform the design of effective interventions to promote increased CS attendance. This would require the translation of the barriers into behaviour change interventions, which should be compared in a randomised controlled trial against interventions known to be effective at improving screening uptake (e.g. [9]). 

This study demonstrates the importance of individual variables in predicting cervical screening uptake. From our sample of women in London, higher levels of household income was the only demographic variable to predict regular attendance. The individual barriers and facilitators which predicted attendance were prioritising cervical screening, memory, and environmental context and resources. Efforts to increase cervical screening attendance should include reminders, decreasing logistical barriers to attendance, and efforts to change attitudes so attending screening is prioritised over other competing demands. Addressing these factors is likely to be an effective way to increase cervical cancer screening uptake, and thus reduce cervical cancer mortality.

## Supplementary Information

**Additional file 1.** 

**Additional file 2.** 

## Data Availability

The datasets used and/or analysed during the current study are available from the Imperial College London Research Data Repository, with https://doi.org/10.14469/hpc/10175.

## Abbreviations

CS: Cervical Screening
TDF: Theoretical Domains Framework
