# Supplementary material for: A population survey on beliefs around cervical cancer screening: determining the barriers and facilitators associated with attendance
Source: BMC Cancer. 2022 May 9;22:522. doi: 10.1186/s12885-022-09529-w (PMC9082843; doi:10.1186/s12885-022-09529-w)
Supplement: Supplementary file 1 — Additional file 1. [file 12885_2022_9529_MOESM1_ESM.docx]

**Additional file 1: survey domains for individual barriers and motivators and their Cronbach’s alpha scores.**

| **Constructs** | **Cronbach’s** **Alpha** | **Items** |
| --- | --- | --- |
| 1. Knowledge of cervical cancer | .720 | Cervical cancer can develop on a woman’s cervix |
|  |  | Cervical cancer can develop and grow with no or few symptoms |
| 2. Knowledge of cervical cancer screening programme | .874 | Women in the UK are offered cervical smear tests as part of cervical screening |
|  |  | Women are invited to cervical screening regularly through the NHS Cervical screening programme |
|  |  | I am aware of the Cervical screening programme |
| 3. Knowledge of benefits of screening | .910 | A smear test can detect cervical cancer in its early stages |
|  |  | If cervical cancer is treated early, survival is more likely |
|  |  | The smear test can save my life |
|  |  | A smear test can detect early changes to the cervix before they turn into cancer |
|  |  | Having regular smear tests can prevent cervical cancer |
| 4. Knowledge of cervical cancer risk factors | .871 | Having many sexual partners |
|  |  | Starting to have sex at a young age (before age 17) |
|  |  | Having a sexual partner with many previous partners |
| 5. Perceived risk | .860 | A woman of my age is unlikely to develop cervical cancer |
|  |  | A woman like me is unlikely to develop cervical cancer |
| 6. Perceived behavioural control | .845 | I have complete control over whether I have a smear test |
|  |  | It is completely up to me whether or not I have a smear test |
| 7. Emotional consequences of potential results | .900 | I am worried about receiving an abnormal test result |
|  |  | I am worried about a cancer diagnosis |
| 8. Value | .825 | Valuable-Worthless (R) |
|  |  | Useless-Beneficial |
|  |  | A Priority-Not Important (R) |
| 9. Belief about test effectiveness/specificity | .574 | Cervical cancer might be missed by a smear test |
|  |  | Cervical cancer might be diagnosed from the smear test when really there is no cancer |
| 10. Reassurance | .869 | A normal smear test gives me reassurance |
|  |  | I feel relieved when I receive a normal test result |
| 11. Previous negative experience | .869 | My previous smear tests have been unpleasant |
|  |  | When I had a smear test, it was worse than I thought it would be |
| 12. Intention | .956 | If/when I am invited for a smear test I intend to attend |
|  |  | If/when I am invited for a smear test I will definitely attend |
| 13. Health priority | .803 | I put my health first |
|  |  | Taking care of my own health is extremely important to me |
| 14. Cervical screening Priority | .945 | If/when invited for cervical screening, I always make an appointment as a priority |
|  |  | Making sure I am up to date with my smear tests is something I prioritise |
| 15. Memory | .730 | I never forget about routine health appointments |
|  |  | When invited in the past, I have never forgotten to make an appointment for a smear test |
| 16. Environmental context and resources | .777 | The distance of the smear test location from my home will affect whether I attend my appointment |
|  |  | Availability of transport (public or personal) to the screening centre might affect me attending my appointment |
|  |  | Being able to get time off work affects whether I can attend health appointments |
|  |  | Being able to find childcare affects whether I can attend health appointments |
| 17. Social norms – descriptive | .775 | My female friends have regular smear tests |
|  |  | The female members of my family have regular smear tests |
| 18. Social norms – injunctive peers | .759 | My friends approve me having cervical smears regularly |
|  |  | The people in my life whose opinions I value would approve of me having regular smear tests |
| 19. Social norms – healthcare professionals | .718 | My GP expects me to attend my smear test appointment when invited |
|  |  | Generally speaking, I tend to do what my GP expects of me |
| 20. Anticipated pain/embarrassment | .832 | I am worried that the smear test will be painful |
|  |  | I am worried that the smear test will be unpleasant |
|  |  | I expect to be very embarrassed during a smear test |
| 21. Behaviour regulation and planning | .936 | I have a clear plan of how I will make time to schedule my smear test |
|  |  | I have a clear plan of how I will make time to attend my smear test |
