# Supplementary material for: A population survey on beliefs around cervical cancer screening: determining the barriers and facilitators associated with attendance
Source: BMC Cancer. 2022 May 9;22:522. doi: 10.1186/s12885-022-09529-w (PMC9082843; doi:10.1186/s12885-022-09529-w)
Supplement: Supplementary file 2 — Additional file 2. [file 12885_2022_9529_MOESM2_ESM.docx]

**Additional file 2**

**Comparison of barriers and motivators between regular attenders and non-regular attenders**

|  | Non-regular attenders | | Regular attenders | | t | df | Sig |
| --- | --- | --- | --- | --- | --- | --- | --- |
|  | Mean | SD | Mean | SD |  |  |  |
| Knowledge of cervical cancer | 5.78 | 1.06 | 5.96 | 1.08 | -1.72 | 459.0 | 0.087 |
| Knowledge of cervical cancer^ screening programme | 6.20 | 0.94 | 6.48 | 0.65 | -3.41* | 239.4 | 0.001 |
| Knowledge of benefits of screening^ | 6.00 | 0.82 | 6.44 | 0.61 | -5.88** | 251.9 | <0.001 |
| Knowledge of cervical cancer risk factors | 4.83 | 1.41 | 4.86 | 1.41 | -0.22 | 459.0 | 0.829 |
| Value^ | 5.40 | 1.17 | 6.47 | 0.68 | -10.57** | 215.4 | <0.001 |
| Cervical Screening Priority^ | 3.72 | 1.58 | 6.28 | 0.95 | -18.78** | 219.7 | <0.001 |
| Planning^ | 3.80 | 1.63 | 5.72 | 1.34 | -12.75** | 272.0 | <0.001 |
| Social norms – descriptive | 4.49 | 1.22 | 4.96 | 1.38 | -3.57** | 459.0 | <0.001 |
| Social norms – injunctive peers | 4.90 | 1.21 | 5.52 | 1.26 | -5.12** | 459.0 | <0.001 |
| Social norms – healthcare professionals^ | 4.92 | 1.20 | 5.91 | 1.10 | -8.61** | 299.7 | <0.001 |
| Memory^ | 4.08 | 1.28 | 5.98 | 1.00 | -16.25** | 261.5 | <0.001 |
| Environmental context and resources^ | 4.31 | 1.28 | 5.15 | 1.49 | -6.34** | 365.3 | <0.001 |
| Perceived risk^ | 5.03 | 1.48 | 5.74 | 1.35 | -5.01** | 297.2 | <0.001 |
| Belief about test effectiveness/specificity^ | 4.42 | 0.96 | 4.26 | 1.24 | 1.50 | 394.7 | 0.135 |
| Anticipated pain/embarrassment^ | 4.90 | 1.39 | 3.77 | 1.72 | 7.60** | 385.4 | <0.001 |
| Previous negative experience^ | 4.53 | 1.57 | 3.61 | 1.76 | 5.73** | 355.7 | <0.001 |
| Emotional consequences of potential results | 4.53 | 1.56 | 4.83 | 1.57 | -1.95 | 459.0 | 0.052 |
| Perceived behaviour control | 5.64 | 1.14 | 5.82 | 1.23 | -1.50 | 459.0 | 0.133 |
| Reassurance^ | 5.50 | 1.15 | 6.35 | 0.80 | -8.27** | 239.5 | <0.001 |
| Health priority^ | 5.06 | 1.23 | 6.00 | 0.89 | -8.54** | 247.1 | <0.001 |
| Intention^ | 4.47 | 1.76 | 6.64 | 0.81 | -14.73** | 193.7 | <0.001 |

Comparison of psychological domains between regular attenders and non-regular attenders

^ Levene’s test for equality of variances was significant, so the test conducted did not assume equal variances.

* p<.01, ** p<.001
